# Study on Quality Prediction of 2219 Aluminum Alloy Friction Stir Welding Based on Real-Time Temperature Signal

**DOI:** 10.3390/ma14133496

**Published:** 2021-06-23

**Authors:** Haijun Wang, Diqiu He, Mingjian Liao, Peng Liu, Ruilin Lai

**Affiliations:** 1 State Key Laboratory of High-Performance Complex Manufacturing, Light Alloy Research Institute of Central South University, Changsha 410083, China; wanghaijun1019@126.com (H.W.); hdqzzp@163.com (D.H.); lmj15874998962@163.com (M.L.); xiangsi0804@163.com (P.L.); 2 Powder Metallurgy Research Institute, Central South University, Changsha 410083, China

**Keywords:** friction stir welding, weld quality prediction, wavelet packet, temperature signal, AA2219-T6

## Abstract

The online prediction of friction stir welding quality is an important part of intelligent welding. In this paper, a new method for the online evaluation of weld quality is proposed, which takes the real-time temperature signal as the main research variable. We conducted a welding experiment with 2219 aluminum alloy of 6 mm thickness. The temperature signal is decomposed into components of different frequency bands by wavelet packet method and the energy of component signals is used as the characteristic parameter to evaluate the weld quality. A prediction model of weld performance based on least squares support vector machine and genetic algorithm was established. The experimental results showed that, when welding defects are caused by a sudden perturbation during welding, the amplitude of the temperature signal near the tool rotation frequency will change significantly. When improper process parameters are used, the frequency band component of the temperature signal in the range of 0~11 Hz increases significantly, and the statistical mean value of the temperature signal will also be different. The accuracy of the prediction model reached 90.6%, and the AUC value was 0.939, which reflects the good prediction ability of the model.

## 1. Introduction

Friction stir welding (FSW) is a kind of solid-state welding method with good efficiency and high weld performance, which is widely used in engineering. Automation and intelligence in the welding process is an important direction for the development of the FSW process. The main focus of this research is how to detect and evaluate the weld quality in the welding process. The traditional methods of FSW inspection and evaluation, such as ultrasonic inspection, X-ray inspection, and coloring inspection, are difficult to implement in the welding process. Therefore, accurate online monitoring of the welding process has become an important focus of research [1,2,3]. Du et al. [4] combined the numerical simulation model with a machine learning algorithm to simulate the maximum shear stress and strain rate in the welding process, studied the influence of the rules on the lifespan of the tool, and built the tool’s life prediction model based on three machine learning algorithms to realize an accurate prediction of tool failure. Verma et al. [5] used rotational speed, traverse speed, and tilt angle as input variables to study the ultimate tensile strength of the welding seam using machine learning methods, such as Gaussian regression (GPR), support vector machine (SVM), and artificial neural network (ANN), and realized the purpose of welding process optimization. Sumesh et al. [6] used the current amplitude signal to establish a direct connection with the weld quality, extracted the statistical features of the original data through data-mining software, established a J48 and random forest algorithm, and reported the classification effect on the weld quality.

FSW is a typical multifactor process. In addition to the process parameters, the size change of the workpiece, the disturbance during welding, and other factors will affect the weld quality. The real-time temperature during welding is an important signal reflecting the welding state. Wedge et al. [7] found that the temperature significantly affects the dimension and distribution of the θ phase in the FSW zone of the 2219 aluminum alloy and the behavior of the weld fracture failure. Shi et al. [8] analyzed the plasticized material flow in the welding process, which showed that the process parameters would affect the material flow, and there is an obvious difference between the temperatures of the advanced side and the retreating side of the welding zone. Samir et al. [9] studied the evolution of the welding temperature and residual stress of the FSW welds by using a thermal-force numerical model. They found that, as the welding speed rose, the residual stress increased. Because FSW is essentially a process of friction heat generation, the welding temperature, particularly the temperature state of the interface between the tools and materials, is of great importance to the quality of the weld. However, in the development of FSW, the relationship between the welding temperature and weld quality, particularly the influence mechanism of the temperature characteristics on the interface between the tools and materials in regard to welding defects, has not been fully established, and there are few reports on the prediction of weld quality using the measured temperature signals [10,11,12]. Here, we aim to establish the relationship between the measured temperature signal and the weld quality by studying the temperature signal characteristics of the interface between the tool and the material.

The 2219 aluminum alloy is a typical representative of Al-Cu alloy with enhanced strength-to-weight ratio and high fracture toughness, which is commonly used in aerospace structures, e.g., rocket fuel tanks. At present, the FSW is the preferred welding method for rocket tanks [7]. In this paper, the FSW process experiment for the 2219 aluminum alloy was carried out under different conditions. The temperature signal of the interface between the tool and material was measured by a thermocouple. We used the wavelet packet method to process the original temperature data, decompose the temperature signal components related to the weld quality, and extract the deep characteristics of the temperature signal. The sampling observation and mechanical property test were performed on the welding seam, and the quality of the joint and the fracture morphology of the sample was observed. The weld quality prediction was studied by the least-square support vector machine (LSSVM) and genetic algorithm.

## 2. Materials and Methods

### 2.1. Experimental Procedure

**Figure 1:**
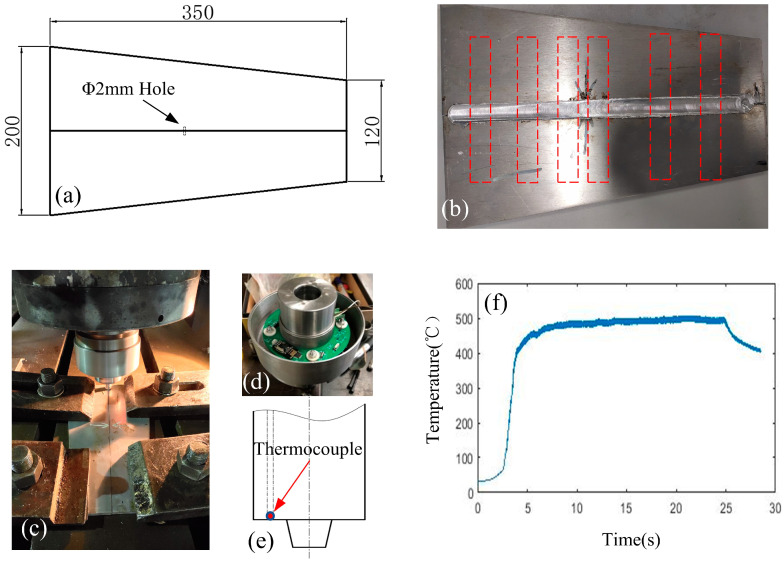
Welding experiment process: (**a**) The main dimensions of the workpiece; (**b**) Welding specimen; (**c**) Temperature signal processing module; (**d**) Friction stir welding experiment; (**e**) Position of temperature measuring point of thermocouple embedded tool; and (**f**): Temperature signal during welding.

The welding material is 2219-T6 aluminum alloy with a thickness of 6 mm, and the size of the plate is shown in Figure 1a. The unequal width design of the plates will change the heat dissipation rate during welding. In order to manufacture welding defects at the designated location and study these defects, a small hole with a diameter of 2 mm and a depth of 5 mm was processed on the joint surface of the plate. When the tool is welding at the hole position, welding defects will be formed because of the sudden loss of material [13,14]. A thermocouple is implanted into the rotating tool to obtain the temperature signal during the welding process. The thermocouple tip is located at the contact position between the tool and the material, as shown in Figure 1e. The signal processing module (see Figure 1d) transmits the temperature signal to the external device, and the sampling frequency is 180 Hz. According to previous research experience, a total of 16 groups of welding experiments were designed for different rotating speeds and traverse speeds and with an appropriate shoulder plunge and tilt angle, as shown in Table 1. The tensile test samples were cut at a given position on the welded seam Figure 1b to observe the welding quality. The fracture morphology was observed by scanning electron microscopy equipment.

### 2.2. Models and Methods

In this paper, an online method for the evaluation of weld quality based on the wavelet packet method, LSSVM, and genetic algorithm is established. As a time-frequency analysis method for the temperature signal, the wavelet packet is used to obtain the temperature signal components of different frequency bands and to extract the temperature characteristic components that reflect the weld quality. LSSVM was used to implement the prediction model of weld quality classification, and the parameters of the model were optimized using the genetic algorithm.

#### 2.2.1. Wavelet Packet Decomposition

When the welding material is extruded and stirred by the high-speed rotating tool, the work done by the tool on the material is asymmetric, so the interface between the tool and the material shows the characteristics of uneven temperature distribution [15,16]. During welding, the temperature is affected by the change in the size of the workpiece section and the accumulation of heat. At the same time, the temperature signal obtained from the high-speed rotating equipment also contains the interference of system noise and white noise [12,17]. Obtaining the characteristics related to the weld quality from the original temperature signal is a task that must be solved. The wavelet packet method can obtain the characteristics of the signal in the time and frequency domains and can decompose the low-frequency and high-frequency signals simultaneously. According to the characteristics and analysis requirements of the signal, the frequency band is adaptively selected to match the signal spectrum. The original signal is decomposed by the low-pass filter and the high-pass filter. These filters are determined by scale function and wavelet function [18,19].

(1) $$\phi (t)=\sqrt{2}\sum_{n}h(n)\phi (2t-n)$$

(2) $$\Psi (t)=\sqrt{2}\sum_{n}g(n)\phi (2t-n)$$

where *φ*(*t*) is the scale function, *Ψ*(*t*) is the wavelet function, *h*(*n*) is the high-pass filter coefficient, and *g*(*n*) is the low-pass filter coefficient.

**Figure 2:**
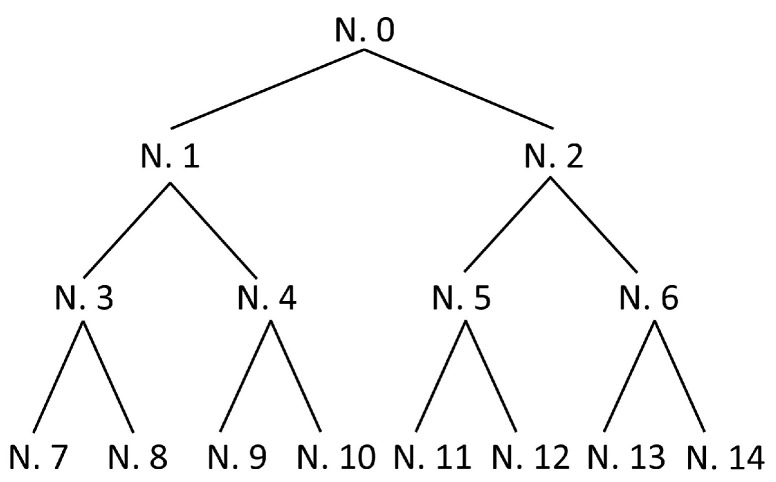
Three-layer wavelet packet signal decomposition tree.

In each step of the temperature signal decomposition, the original signal is divided into an approximate signal of low frequency and a detailed signal of high frequency when the wavelet packet method is used to extract features. If the original signal is decomposed into the *m* layer, *2^m^* wavelet packet coefficients are finally obtained, and the original signal is decomposed into *2^m^* frequency bands components [20]. Figure 2 presents the schematic diagram of the three-layer wavelet packet decomposition tree.

To study the mapping law between temperature and weld quality, we extracted the energy characteristics of the temperature signal component. For example, the signal set obtained after the reconstruction of the wavelet packet coefficient signal of the *m* layer is [*D*_*m*0_, *D*_*m*1_, … *D_m_*_(2_*^m^*_−1__)_] and the energy of the reconstructed signal is [21]:

(3) $$E_{mn}=\sum_{k=1}^{s}{\left|D_{mnk}\right|}^{2}$$

where *n* represents the sequence number of the wavelet packet coefficients and *s* represents the number of points of each wavelet packet coefficient in the *m* layer. The wavelet function used in this paper is the fourth order Daubechies wavelet, and the original temperature signal is decomposed using the three-layer wavelet packet.

#### 2.2.2. GALSSVM Model

**Figure 3:**
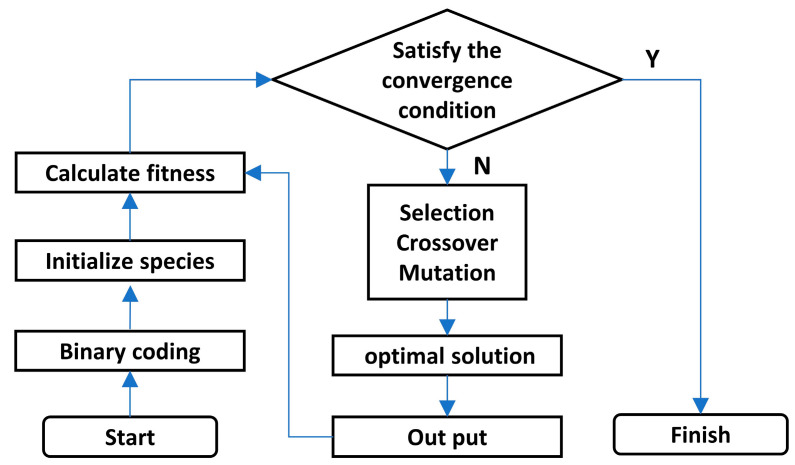
Genetic optimization algorithm workflow.

The FSW process is a highly nonlinear process with multiple-input influence, and it is difficult to establish the relationship between input and output directly through mathematical formulas [22,23]. The machine learning method uses the powerful data processing ability of a computer to solve complex problems and becomes a feasible way to realize the welding prediction [24,25]. The SVM is a linear classifier that tries to find a hyperplane to separate sample data by the highest margin. For nonlinear problems, the integral operator kernel function is used to map the data into a high-dimensional eigenspace. The SVM calculation can be transformed to solve a convex quadratic programming problem [26,27]. Here, we adopted the LSSVM algorithm, which replaces the inequality constraints of the original quadratic programming in SVM with an equality constraint to reduce the computation consumption and improve the learning performance. The solution model function of LSSVM can be expressed as [28,29]:

(4) $$f(x)=\omega^{T}\phi (x)+b$$

where *φ*(**x**) is the mapping function, **x** is the input variables, **ω** is the weight vector, and *b* is the bias value. The quadratic programming problem for LSSVM is given as:

(5) $$\begin{array}{l} \min J(\omega ,e)=\frac{1}{2}\omega^{T}\omega +\frac{1}{2}C\sum_{k=1}^{n}e_{k}^{2}\\ s.t.\;y_{k}=\omega^{T}\phi (x_{k})+b+e_{k},k=1,2,\ldots ,n \end{array}\}$$

where *C* is the penalty factor and *e_k_* is the error between the predicted and the actual output. Using the Lagrange multiplier method and kernel function, the nonlinear regression model is transformed as [27]:

(6) $$f(x)=\sum_{i=1}^{n}\alpha_{i}K(x,x_{i})+b$$

where *α_i_* is the Lagrange multiplier and *K*(*x, x_i_*) is the kernel function that satisfies the Mercer condition. The kernel function is the key to the LSSVM algorithm. Its function is to transform the data into high-dimensional space. We adopted the Gaussian kernel function in this paper, and its expression is given in Equation (7) [30]:

(7) $$K(x,x_{i})=\exp\left\{\gamma {\left\|x-x_{i}\right\|}^{2}\right\}$$

where *γ* is the kernel parameter, and *γ* and *C* are the crucial parameters for the LSSVM model. In this paper, the genetic optimization algorithm (GA) was used to optimize the value of *γ* and *C*. The optimization process mainly includes binary coding of the parameters, establish the initial population, takes the accuracy of the prediction model as the fitness function, and then selects, crosses, and mutates until the optimized parameters are obtained. The workflow of the GA algorithm is shown in Figure 3.

According to the requirements of the engineering application, we evaluated the weld quality based on the tensile strength of the joints. According to the coefficient *σ* of the 2219 aluminum alloy substrate (420 MPa), the weld quality was divided into three types as shown in Table 2. Here, the one versus rest (OVR) method was used to solve the tri-classification prediction problem. We constructed three LSSVM models, of which the *j* one is used to judge whether the sample belongs to the *j* class or not. The temperature signal characteristics of the welding process are taken as the input variables for the prediction model. At the same time, because the process parameters are the key factors in determining the weld quality, we also take two variables, rotational speed and traverse speed, as the input for the model.

## 3. Results and Discussion

### 3.1. Experimental Results

**Figure 4:**
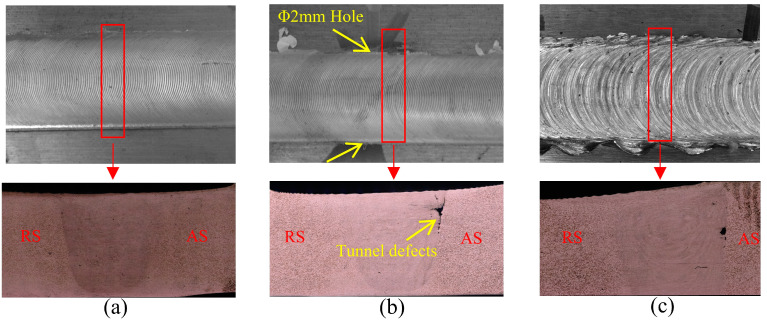
Weld quality of different joints: (**a**) Perfect joint (rotational speed: 1150 r/min, traverse speed: 300 mm/min); (**b**) Joint after sudden perturbation (rotational speed: 1150 r/min, traverse speed: 300 mm/min); and (**c**) Welding joint with abnormal workmanship (rotational speed: 1300 r/min, traverse speed: 100 mm/min).

In this paper, we conducted welding experiments under different process conditions, observed the weld samples, evaluated the weld quality by tensile testing, and studied the typical characteristics of the welding defects. The weld quality of different joints is shown in Figure 4.

**Figure 5:**
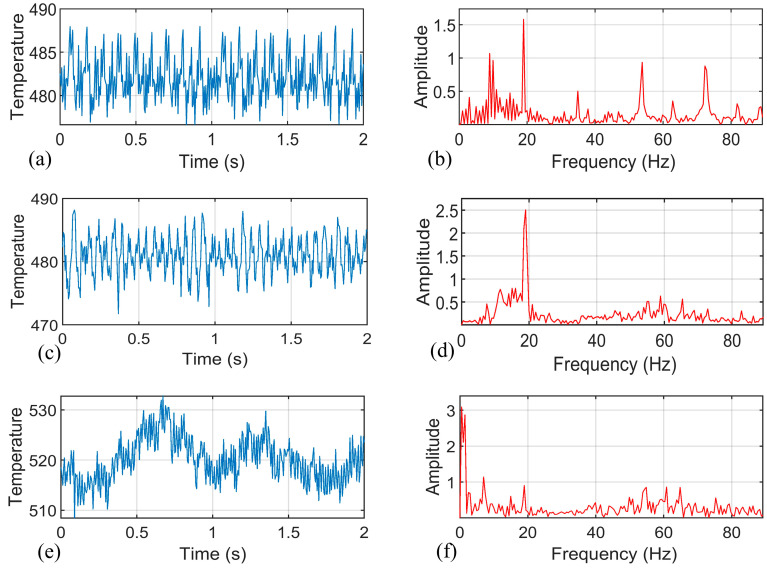
Raw data of temperature signal: (**a**) Temperature signal of the perfect joint; (**b**) Spectrum of the temperature signal of the perfect joint; (**c**) Temperature signal after sudden perturbation; (**d**) Spectrum of temperature signal after sudden perturbation; (**e**) Temperature signal of the joint with abnormal workmanship; and (**f**) Spectrum of the temperature signal of the joint with abnormal workmanship.

**Figure 6:**
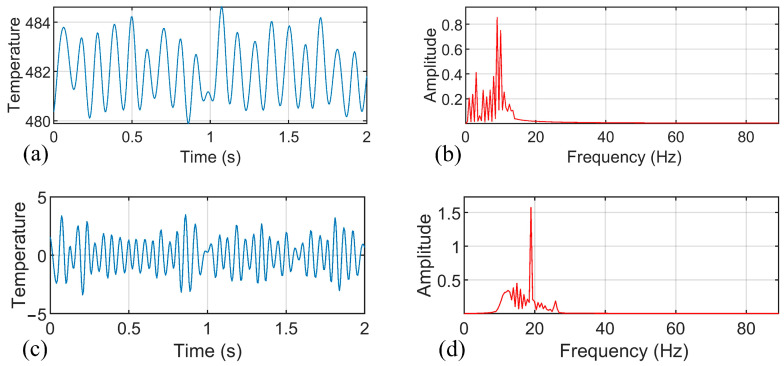
Temperature signal component of perfectly formed welds: (**a**) Temperature signal component decomposed by wavelet packet at node 7; (**b**) Spectrum of temperature signal component at node 7; (**c**) Temperature signal component decomposed by wavelet packet at node 8; and (**d**) Spectrum of temperature signal component at node 8.

**Figure 7:**
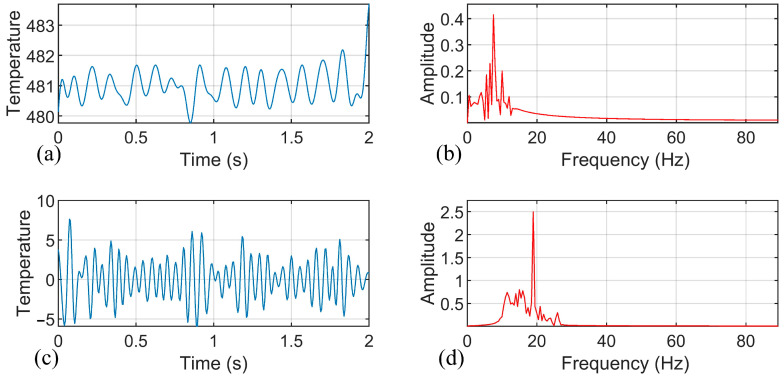
Temperature signal component of welds with tunnel defects formed after sudden perturbation: (**a**) Temperature signal component decomposed by wavelet packet at node 7; (**b**) Spectrum of temperature signal component at node 7; (**c**) Temperature signal component decomposed by wavelet packet at node 8; and (**d**) Spectrum of temperature signal component at node 8.

**Figure 8:**
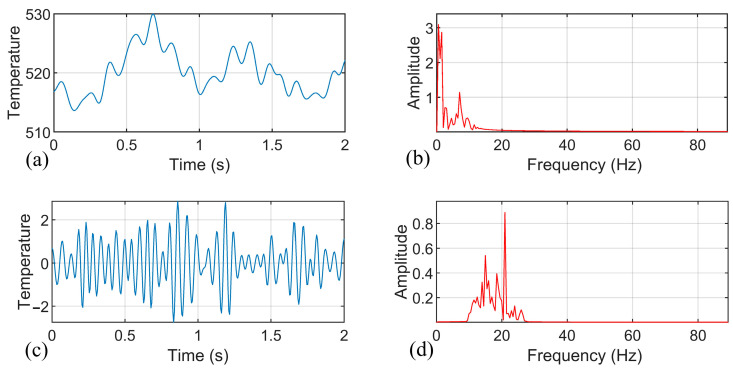
Temperature signal component of welds with improper process: (**a**) Temperature signal component decomposed by wavelet packet at node 7; (**b**) Spectrum of temperature signal component at node 7; (**c**) Temperature signal component decomposed by wavelet packet at node 8; and (**d**) Spectrum of temperature signal component at node 8.

**Figure 9:**
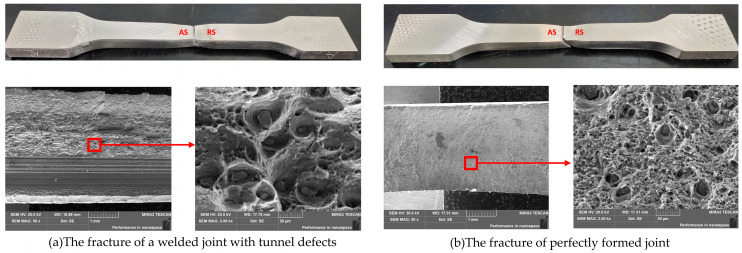
Fracture morphology observation of the tensile specimen.

The temperature signal analysis are shown in Figure 5, Figure 6, Figure 7 and Figure 8 (n.b. Different scaling). The signal with a duration of 2 s was intercepted at the observation position of the weld for research. The spectrum of the signal shows that the original temperature data is composed of components of multiple frequency bands. According to Figure 5a,b, the temperature signal corresponding to the well-formed weld (rotational speed: 1150 r/min, traverse speed: 300 mm/min) was observed, there were high-frequency noise components on the spectrum, and there was an obvious peak component near the tool rotation frequency (19 Hz), with an amplitude of about 1.6. According to Figure 5c,d, when the welding process reached the position of the hole, the tunnel defect was formed because of the sudden material loss, as shown in Figure 4c. The main change in the temperature signal in the figure was that the amplitude of the peak component near the tool rotation frequency increased to 2.5. The fracture morphology of joints with tunnel defects and perfectly formed joints was observed (see Figure 9). The fracture locations were all located near the forward side of the weld nugget, and the joints with tunnel defects derived cracks from the position of the hole and eventually fractured. The results of the scanning electron microscopy (SEM) showed that, compared with the fracture morphology of the perfect joint, the fracture morphology near the tunnel defect had larger dimples, and the second phase particles of larger size could be seen inside. In the weld core area, the material underwent dynamic recrystallization and formed a fine grain structure because of the strong stirring and crushing effect of the tool [31]. According to the Zener–Holloman model theory, the grain size is positively correlated with the strain rate [32]. It can be considered that, for welded joints with tunnel defects, the extrusion and friction on the material behind the shoulder is insufficient when the tool is moving forward, and the heat generation is inadequate as well. Therefore, the temperature signal spectrum shows a peak change near the tool rotation frequency.

According to Figure 5e,f, when the welding process parameters are inappropriate (rotational speed 1300 r/min, welding speed 100 mm/min), the temperature signal showed a higher amplitude component in the low-frequency band (0~10 Hz). Meanwhile, the statistical mean value of the temperature signal also changed significantly, from 482 °C for the well-formed weld to 519 °C. According to the appearance of the quality of the weld surface, the suitable welding process surface is a uniform grain, as shown in Figure 4a, while the unsuitable process parameters will produce uneven grain features, showing the characteristics of alternate deep and shallow paths, as shown in Figure 4c.

According to the experimental results, when weld quality problems are caused by inappropriate welding process or a sudden perturbation in the welding process, the main changes in the temperature signals are the amplitude of the components in the low-frequency band (0~10 Hz), the amplitude of the components near the tool rotation frequency, and the statistical mean temperature. Therefore, we use the three-layer wavelet packet method to further study the original. The three-layer wavelet packet decomposition tree is shown in Figure 2, where the signals of nodes 7 and 8 correspond to the signal components in the frequency band of 0~11 Hz and 11~22 Hz, respectively. Node 8 is the frequency band component of the rotational speed, reflecting the temperature characteristics of the uneven distribution of the contact interface between the tool and the material. The temperature signals of the different welding conditions were decomposed by the wavelet packet, and the signal components of nodes 7 and 8 were reconstructed. Additionally, Figure 6 shows the temperature signal component of the perfectly formed welds, Figure 7 shows the temperature signal component of the welds with tunnel defect formation after sudden perturbation, and Figure 8 shows the temperature signal component of welds using an improper process. The calculated signal energy values were used as the input variables of the weld quality prediction model.

### 3.2. Predictive Model Results

We conducted FSW experiments under different process conditions, evaluated the welding quality through tensile testing, and obtained 96 groups of test data samples (partial data are shown in Table 3). The energy value obtained by the wavelet packet method is used as the influencing variable to evaluate the weld quality. After the homogenization, the energy data are used in the GA-LSSVM prediction model. The input variables of the model include the statistical mean value of the welding temperature signal, component signal energy at nodes 7 and 8, rotational speed, and traverse speed.

**Figure 10:**
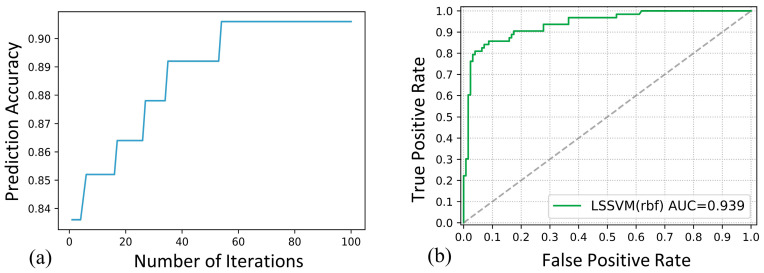
Prediction model performance: (**a**) Model accuracy after genetic optimization and (**b**) Forecast model ROC curve.

To study the prediction performance of the model and ensure the appropriate number of test sets and training sets, we used the method of five-fold cross validation. The original data set was divided into five groups, one of which was used as the test set and the others as the training sets. A total of five evaluation results were obtained, and the prediction performance of the final model was obtained by taking the average value of the 5 results [33]. After 100 iterations of the genetic optimization algorithm, the optimal model parameters *γ* and *C* are 8.3 and 2.1, respectively, and the accuracy of the model prediction reached 90.6%, as shown in Figure 10a. Figure 10b shows the ROC curve of the model under different decision thresholds [34], and the area under the curve (AUC) reaches 0.939, which reflects the model’s good prediction ability.

## 4. Conclusions

**Figure 11:**
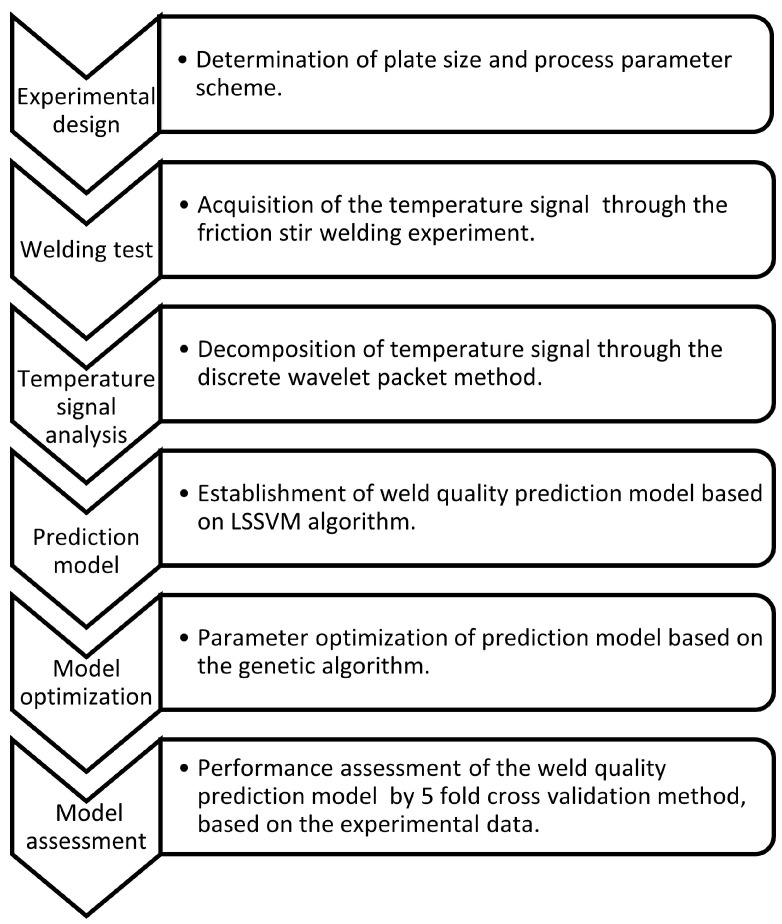
Process and approaches of constructing FSW quality prediction model.

The real-time temperature signal obtained from the interface between the tools and materials can play an important role in the online monitoring of weld quality. The process and approaches to constructing the FSW quality prediction model are shown in Figure 11. The results of the FSW experiments show that the welding quality problems caused by different factors present different characteristic changes in the temperature signal. The conclusions of this paper are as follows:

1. Tunneling defects are formed when sudden perturbations are experienced during welding. In the feed direction, plastic flow and heat generation of the material behind the tool is insufficient. The welding temperature signal shows the amplitude variation of the signal component near the tool rotation frequency.
2. When inappropriate process parameters are used, welding defects, such as large flints and uneven surface texture, will be caused, and changes in low-frequency components (0~10 Hz) and statistical mean characteristics will be shown in the welding temperature signal.
3. The low-frequency component of the original temperature signal and the frequency component containing the rotation frequency of the tool were extracted using the three-layer wavelet packet method, and the energy value of the component signal was obtained. The characteristics of these temperature signals played an important role in improving the efficiency of the weld quality identification. Using the extracted temperature component signal energy, statistical mean temperature, rotational speed, and welding speed as input variables, the prediction accuracy of the weld quality classification prediction model established by the GA-LSSVM algorithm can reach 90.6%, and the AUC value is 0.939.

## Figures and Tables

**Table 1:** Weld conditions.

Parameter	Rotation Speed (r/min)	Traverse Speed (mm/min)	Shoulder Plunge (mm)	Tilt Angle (°)
Value	850; 1000; 1150; 1300	100; 200; 300; 400	0.1	1.5

**Table 2:** Definition of welding performance types.

Type	Type 1	Type 2	Type 3
Coefficient of strength	*σ* ≥ 75%	65% < *σ* < 75%	*σ* ≤ 65%

**Table 3:** Test data of partial welds.

Rotational Speed (r/min)	Traverse Speed (mm/min)	Mean Temperature (°C)	Energy 7	Energy 8	Tensile Strength (MPa)	Type
850	100	479	0.24	0.76	264.3	3
1000	100	512	0.36	0.13	318.2	1
1150	100	518	0.11	0.62	296.3	2
1300	100	529	0.15	0.89	259.6	3
850	200	485	0.23	0.24	205.5	3
1000	200	510	0.86	0.16	327.3	1
1150	200	512	0.92	0.14	328.3	1
1300	200	513	0.35	0.24	317.2	1
850	300	528	0.44	0.68	205.6	3
1000	300	489	0.14	0.84	297.3	2
1150	300	493	0.21	0.67	336.2	2
1300	300	511	0.38	0.14	316.3	1
850	400	493	0.29	0.45	302.6	2
1000	400	479	0.76	0.24	264.3	3
1150	400	485	0.83	0.16	234.9	3
1300	400	506	0.08	0.24	319.7	1

## Data Availability

No new data were created or analyzed in this study. Data sharing is not applicable to this article.
